# Assessing groundwater denitrification spatially is the key to targeted agricultural nitrogen regulation

**DOI:** 10.1038/s41598-024-55984-9

**Published:** 2024-03-06

**Authors:** Birgitte Hansen, Jens Aamand, Gitte Blicher-Mathiesen, Anders V. Christiansen, Niels Claes, Tommy Dalgaard, Rasmus R. Frederiksen, Brian H. Jacobsen, Rasmus Jakobsen, Anders Kallesøe, Hyojin Kim, Julian Koch, Ingelise Møller, Rasmus B. Madsen, Stefan Schaper, Peter B. E. Sandersen, Denitza D. Voutchkova, Irene Wiborg

**Affiliations:** 1 Department of Geochemistry, Geological Survey of Denmark and Greenland - GEUS, Øster Voldgade 10, 1350 Copenhagen K, Denmark; 2 Department of Ecoscience, Aarhus University, C.F. Møllers Allé, 8000 Aarhus C, Denmark; 3 Department of Geoscience, Aarhus University, Høegh-Guldbergs Gade 2, 8000 Aarhus C, Denmark; 4 Department of Agroecology, Aarhus University, Blichers Allé 20, 8830 Tjele, Denmark; 5 Department of Food and Resource Economics, University of Copenhagen, Rolighedsvej 23, 1958 Frederiksberg C, Denmark; 6 Department of Near Surface Land and Marine Geology, Geological Survey of Denmark and Greenland - GEUS, Universitetsbyen 81, Building 1872, 8000 Aarhus C, Denmark; 7 Department of Hydrology, Geological Survey of Denmark and Greenland - GEUS, Øster Voldgade 10, 1350 Copenhagen K, Denmark; 8 Department of Management, Aarhus University, Fuglesangs Allé 4, 8210 Aarhus V, Denmark; 9 SEGES Innovation, Agro Food Park 15, 8200 Aarhus N, Denmark

**Keywords:** Environmental sciences, Environmental social sciences

## Abstract

Globally, food production for an ever-growing population is a well-known threat to the environment due to losses of excess reactive nitrogen (N) from agriculture. Since the 1980s, many countries of the Global North, such as Denmark, have successfully combatted N pollution in the aquatic environment by regulation and introduction of national agricultural one-size-fits-all mitigation measures. Despite this success, further reduction of the N load is required to meet the EU water directives demands, and implementation of additional targeted N regulation of agriculture has scientifically and politically been found to be a way forward. In this paper, we present a comprehensive concept to make future targeted N regulation successful environmentally and economically. The concept focus is on how and where to establish detailed maps of the groundwater denitrification potential (N retention) in areas, such as Denmark, covered by Quaternary deposits. Quaternary deposits are abundant in many parts of the world, and often feature very complex geological and geochemical architectures. We show that this subsurface complexity results in large local differences in groundwater N retention. Prioritization of the most complex areas for implementation of the new concept can be a cost-efficient way to achieve lower N impact on the aquatic environment.

## Introduction

The human interference with the nitrogen (N) cycle has exceeded the planetary boundaries^1–3^ by polluting air and water, with implications for other planetary boundaries, such as biodiversity, livelihoods, and human health^4^, e.g. through consumption of nitrate-containing drinking water^5–8^. For more than three decades, many European countries with intensive farming, including Denmark, have successfully lowered the level of N pollution in the aquatic environment^9,10^ by introducing different mitigation measures aimed at agricultural regulation and management as well as wastewater treatment^11^. Protection of the aquatic environment is enforced by national and European Policy initiatives such as the overarching Water Framework Directive and the amended Groundwater and Nitrates Directives (Nitrates Directive, 1991/696/EC; Water Framework Directive, 2000/60/EC and Groundwater Directive, 2006/118/EF). According to the Nitrates Directive, nitrate vulnerable zones is defined as the areas of land that drain into polluted waters or waters at risk of pollution and which contribute to nitrate pollution. Many of the European countries including Denmark has designated the whole territory as a nitrate vulnerable zone demanding establishment of action programmes or plans in the implementation of the Nitrates Directives.

The greatest effects were achieved with the first Danish action plans targeting agriculture (c. 1985–2000), but in the last decades (c. 2000–2020) the effects have levelled out^10,12,13^, making it more difficult to further lower the N load to the aquatic environment. Therefore, there is a need to resolve this worldwide dilemma by developing methods for additionally lowering the environmental N impact, with minimal consequences for crops and livestock production^14^. Implementing successively stricter national agricultural N measures, such as lower levels of N application to meet the demands of the EU water directives^14,15^, is costly. Almost ten years ago, cost-efficient targeted N regulation of agriculture was suggested as a way to further reduce the impact on Danish groundwater, surface waters and marine waters^16^. Several initiatives have been taken, and the latest Danish political green transition agreement (Oct. 2021) includes plans for a substantial annual reduction of up to 6500 tons N (c. 50% of the total N reduction demands) to the Danish coastlines by means of targeted N regulation^17^.

**Figure 1:**
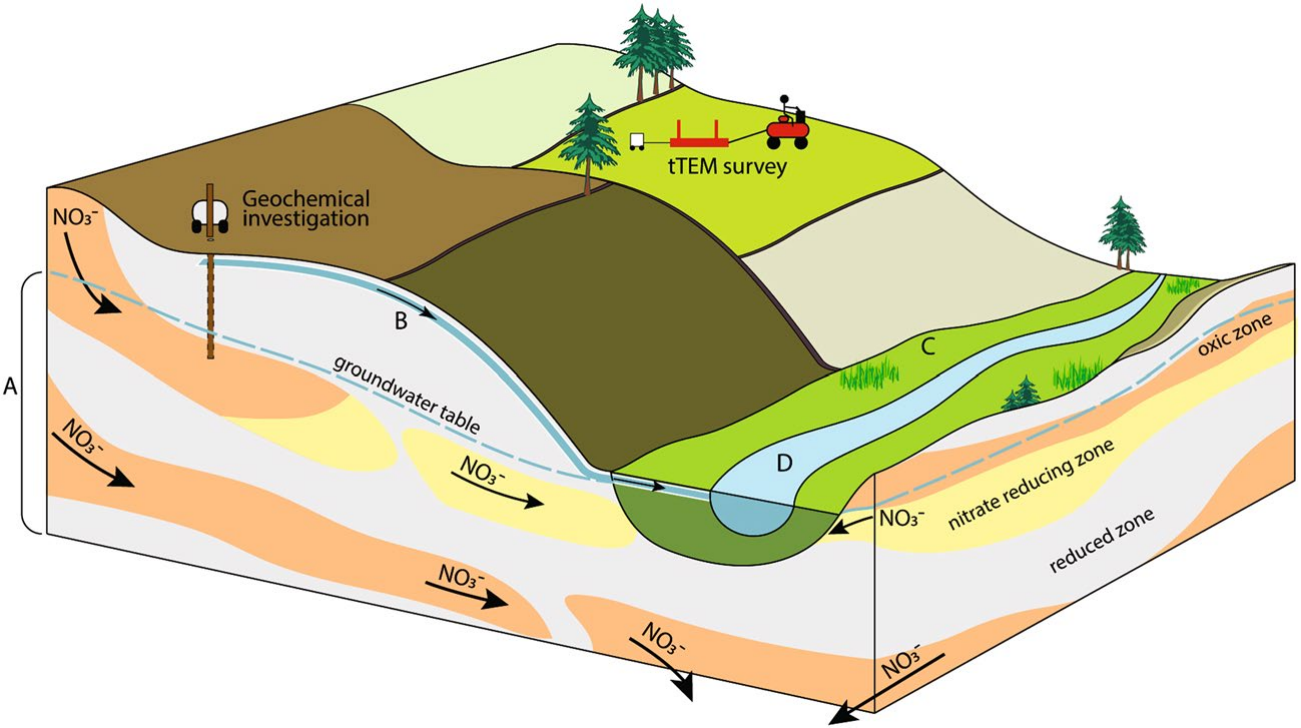
Schematic block diagram of the important pathways and zones for N retention in the landscape and subsurface. (**A**) The groundwater zone, (**B**) the unsaturated zone, incl. root zone, (**C**) the riparian zone and (**D**) the surface water system^34^.

The purpose of targeted N regulation is to limit mitigation measures to the fields where the effects of the measures will be the highest to reduce overall costs. This can be done by adjusting the local N regulation of a field to the field-specific groundwater N retention, and at the same time evaluate N retention in other parts of catchment as shown in Fig. 1. The groundwater N retention is defined as the natural denitrification potential in groundwater, which depends on the water pathways and the N reduction rates and capacities of the groundwater media along the flowpath to the recipient. The N retention mapping aims to delineate robust fields with a high groundwater N retention, and vulnerable fields with a low groundwater N retention. Thus, the purpose of targeted N regulation is then to focus on the vulnerable fields where N leaching should be lowered, e.g., by using more catch crops, set-aside or by lowering the N norms (maximum fertilization level) of specific crops. In this way, targeted N regulation aims at obtaining more sustainable agricultural production with lower environmental impact on groundwater and surface waters.

Possibilities for the implementation of targeted N regulation in agriculture at different scales for protection of surface waters have been the focus of several research projects by building knowledge on the groundwater transport and denitrification potential of nitrate at local^18,19^, catchment^20,21^, national^22,23^, and regional^24^ scales, including assessments of uncertainties^25^. Comparable methods for targeted groundwater protection have been developed based on nitrate vulnerability assessments of the groundwater aquifer used for drinking water production. Since 2000, detailed mapping methods for nitrate vulnerability assessments of aquifers has been developed and implemented in Denmark^26^, recently adapted to the more common used DRASTIC method^27^ originally developed by the US Environmental Protection Agency^28^. A simpler version of DRASTIC have recently been applied and harmonized for different European countries^29^.

In this paper, we present findings from a newly developed concept called N-MAP^30^ from the MapField project (http://mapfield.dk/; Innovation Fund Denmark), resulting in high-resolution groundwater N retention maps (1 hectare scale) for selected catchments with aquifers in Quaternary deposits. These maps are based on modelling of nitrate leaching from the fields, and high-resolution geophysical and geochemical mapping of the subsurface together with geostatistical modelling of hydrostratigraphy, redox zones, and denitrification rates. We hypothesise that the complexity of both geology and redox conditions to a large extent determine the groundwater N retention in the subsurface. The aim of this paper is (1) to demonstrate the value of groundwater N retention maps for targeted N regulation with a high degree of variation in N retention in different Quaternary geological settings, and (2) to propose a prioritisation tool for selecting the areas most suitable for targeted N regulation, and justifying detailed mapping, due to a high complexity of the subsurface geology and geochemistry.

## Groundwater N retention

N retention in the aquatic environment is defined as the natural removal of reactive nitrogen by biogeochemical redox transformations to nitrous oxide (N_2_O) or to inactive nitrogen (N_2_). In a catchment, N retention can mainly take place in (A) the groundwater zone, (B) the unsaturated zone incl. the root zone, (C) river valleys and low-lying areas, and (D) streams and lakes, as illustrated in Fig. 1. The capacities for N retention by denitrification in the groundwater zone are determined by the amounts of the reactive compounds and reduction potentials. Reduction is microbiologically mediated and rates are determined by the reactivity of the electron donors in the geological layers, such as Fe minerals, sulphides e.g., pyrite, or organic matter^31,32^.

In this paper, the focus is on N retention in the groundwater, i.e., zone A shown in Fig. 1, where a high degree of N retention often takes place. The total N retention in a catchment is defined as the difference between the leaching of nitrate from the root zone of the fields and the transport of total nitrogen at the stream outlet from the catchment^23^. In Denmark, national modelling has shown that N retention in the groundwater amounts to *c.* 65% (*c.* 0.125 Tg N per year) of the N leaching from the root zone (*c.* 0.192 Tg N per year) for the period 1990–2010^23^. It is also estimated that the N retention in the groundwater and the unsaturated zone (Fig. 1A,B) amounts to *c.* 88%, and N retention in river valleys, lowlands, streams and lakes reaches *c.* 12% of the total annual N retention to Danish coastal catchments for the period 1990–2010^23^.

## Potentials for targeted N regulation

Six small catchments of *c.* 500–3,000 hectares in size have been investigated in detail using the N-MAP concept^33^. The concept consists of four phases: (1) compiling existing data and information, (2) high resolution geophysical mapping, geochemical sampling, and geostatistical modelling, (3) root zone modelling, groundwater flow modelling and particle tracking, and N retention estimation, and (4) dissemination of N retention maps to stakeholders such as farmers and authorities to facilitate the process of implementing locally targeted N reduction requirements.

**Figure 2:**
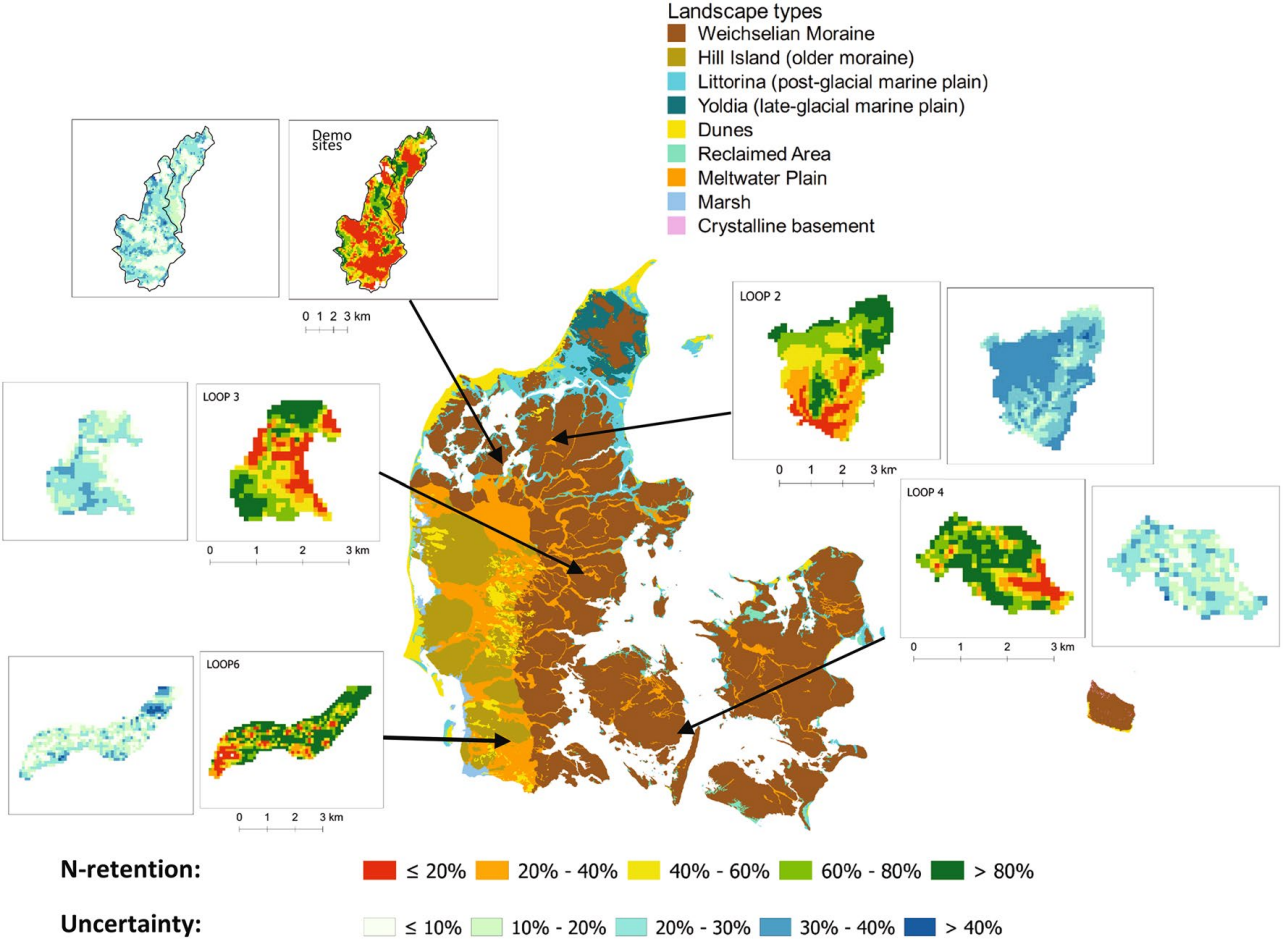
Danish landscape types^65^, and detailed (100 × 100 m) groundwater N retention maps for six selected catchments with different types of complex Quaternary aquifers in Denmark generated with QGIS v. 3.22.10 Bialowieza^66^. The Demo sites consists of two catchments.

Detailed groundwater N retention maps including uncertainty estimates have been produced at 100 × 100 m resolution (one hectare) for the six study catchments (Fig. 2) based on 500 groundwater model realisations. The uncertainty is calculated based on the variation in N retention estimates between the realisations from the geostatistical modelling, and reflects the uncertainty in the knowledge about model structures and parameters such as hydrostratigraphy, redox units, hydraulic conductivities, storage coefficients, and denitrification rates^33^. Generally, the uncertainty increases with higher resolution of the N retention maps^20,25^. The high degree of spatial variation in the N retention for each of the six catchments indicates a high potential for targeted N regulation within the catchments. For example, areas with high N retention > 80% (green areas) and low N retention ≤ 20% (red areas) vary within the catchments. permanent grassland, or set-aside of agricultural land can be focused to the areas with low retention. On the other hand, fields or areas with high groundwater nitrogen retention can continue with a high level of agricultural production and fertilisation if a low N retention due to direct surface runoff or drainage is negligible. Implementation of the N-MAP concept is thus particularly relevant in catchments where N retention in groundwater varies between fields and where detailed data are needed to increase the certainty of N retention estimates at the sub-catchment level.

## Subsurface complexity

At the study sites, agriculture occupies *c*. 65–99% of the catchment areas and takes place on sandy to clay rich soils with dairy, pig or arable farming (Table 1).

**Table 1:** Characteristics of the six study catchments with aquifers in Quaternary deposits where two catchments are combined in the description of the Demo sites.

Name	Soil type	Main type of agriculture	Landscape type^38^	Quaternary deposits^38^	Redox complexity*	Geological complexity^33,37^
LOOP2	Sandy	Dairy and pig farming	Ice marginal hills and outwash plains	Tills, meltwater sands and clays and postglacial deposits in lowlands	Low: (1–4 m)	High: Glacial tectonic deformation due to weight-relief from the ice
LOOP3	Sand-mixed clays	Arable and dairy farming	Hilly ice marginal glacial landscape with erosion valleys	Tills, meltwater sands and clays, dislocated Eocene clays, and postglacial deposits,	Low to medium: (4–13 m)	Medium high: Glacial tectonic folded layers, and buried valleys
LOOP4	Sand-mixed clays	Arable farming	Dead ice glacial landscape, moraine and kame hills	Clay tills, meltwater clays and sand, aeolian sand, and postglacial deposits in lowlands	Low to medium: (4–11 m)	Moderate to high: Glacial deformation and deep faults
LOOP6	Sandy	Dairy farming	Outwash plains with small remnants of glacial hill-islands	Meltwater sands and clays, clay hills, and scattered postglacial deposits in lowlands	Low to medium high: (1–16 m)	Low to moderate: Deep faults, glacial erosion and deformation
Demo sites	Sandy and sandy clays	Dairy, pig and arable farming	Moraine landscape with erosion valleys influenced by underlying salt diapir	Clay tills, meltwater sands and clays, and postglacial deposits in lowlands	Medium high: (16–18 m)	Moderate to high: Crosscutting buried valleys both large and deep, and narrow and small

*The redox complexity is defined and assessed based on the distance (shown in parentheses in m) between the first redox interface (FRI) and the depth to nitrate containing groundwater–the larger the distance the higher the redox complexity.

The groundwater aquifers are placed in Quaternary deposits under varying landscape types, mainly ice marginal hills and outwash plains with occurrence of erosional valleys (Table 1). The sediments range from tills, meltwater sand and clays to postglacial organic-rich deposits, and aeolian sand.

The complexity of the redox conditions has been determined by analysing the sediment colour changes and water chemistry in boreholes^34,35^. A first redox interface (FRI) is defined as the first interface between oxic and reduced lithological layers determined based on colour changes of the sediment. In Denmark, on a national scale, the depth to the FRI was obtained from the simulations by Koch et al.^22^ using a machine learning method (“Random Forest”) including 15 000 observations of colour changes^35^. Analogous, a map of the depth to nitrate containing groundwater has been modelled using the same ML framework as used for FRI but trained against water chemistry data. The redox complexity at a given location is defined and assessed by evaluating the distance between the FRI and the depth to nitrate containing groundwater (top of screen) and is given a value corresponding to this distance. The larger the distance the higher the redox complexity. At the study sites, the redox complexity varies from low (1–4 m) to medium high (16–18 m) where low redox complexity represents a simple one-redox interface case with agreement between data on colour changes and the occurrence of nitrate-containing water. The sites with a higher redox complexity represent cases where colour descriptions in boreholes reveal several shifts in redox conditions with a relative long distance (up to more than 25 m) between the FRI and the depth to nitrate-containing groundwater. These complex redox conditions have developed due to historic infiltration of oxidants (oxygen and nitrate), different reactivity rates of the geological media with reducing minerals/agents (pyrite and carbon) and varied local to regional hydrogeological transport pathways of oxidants (Table 1).

The complexity of the geology is therefore also important for the redox complexity. The complexity of the geology has been evaluated by analysing landscape types, geomorphology, geological structures and lithology from geophysical surveys and borehole descriptions^36^. At the study sites, planar deposits, such as sandy outwash plains, represent low complexity. On the other hand, deposits that are affected by e.g., glaciotectonic deformation (thrusted and folded layers, and geological sand windows defined as layers with a high risk of pollutant transport from the surface to the aquifers), deep faults, and deep erosions (buried tunnel valleys) represent different degrees of geological complexity (Table 1).

## Variation in groundwater N retention

Each of the catchments have a very distinct groundwater N retention pattern with high N retention areas (green > 80%) and low N retention areas (red ≤ 20%) as seen in Fig. 2. These patterns reflect the complexity of the subsurface with different local hydrogeological flow paths and redox zones with different denitrification rates. However, similarities between the catchments are also seen by a generally lower groundwater N retention in the downstream areas close to the outlet. These downstream areas with low groundwater N retention probably exist due to short transport distances in oxic or anoxic nitrate reducing layers with a low denitrification rate. A patchy appearance of N retention is seen in the glacial outwash plains (e.g., in LOOP2 and LOOP6; Fig. 2) caused by local occurrences of postglacial organic-rich deposits with a high denitrification rate resulting in a high N retention. Low N retention is also seen in areas with near surface oxic sandy layers with no denitrification potential, and very undulating and incoherent redox structures. In Quaternary deposits, these complex structures are found in several distinct types of deposits and structures such as thrusted layers, geological windows, infills in buried valleys or otherwise heterogenous geological settings^35,37,38^. In addition, low N retention is observed in groundwater recharge areas where oxic or nitrate reducing anoxic layers have hydraulic connection to the streams. Possibilities for high N retention are found in homogeneous geological settings with horizontal planar geological layers and near-surface coherent redox interfaces for example as seen in outwash plains^37^.

## Prioritisation of areas for targeted N regulation

A full implementation of the N-MAP concept on all Danish fields is an investment estimated to *c.* €470 M. based on a hectare cost of *c.* €160. This investment, in conjunction with targeted regulation, will not yield an equal return everywhere. The highest benefits are in areas with the largest reduction requirements and where targeted N retention is the most cost-efficient way forward compared to general regulation requirements on all fields. As explained, a high potential for targeted regulation is expected to arise from a large difference in the groundwater N retention between nearby fields due to a high subsurface complexity affecting the pathways and denitrification of percolating nitrate in groundwater.

**Figure 3:**
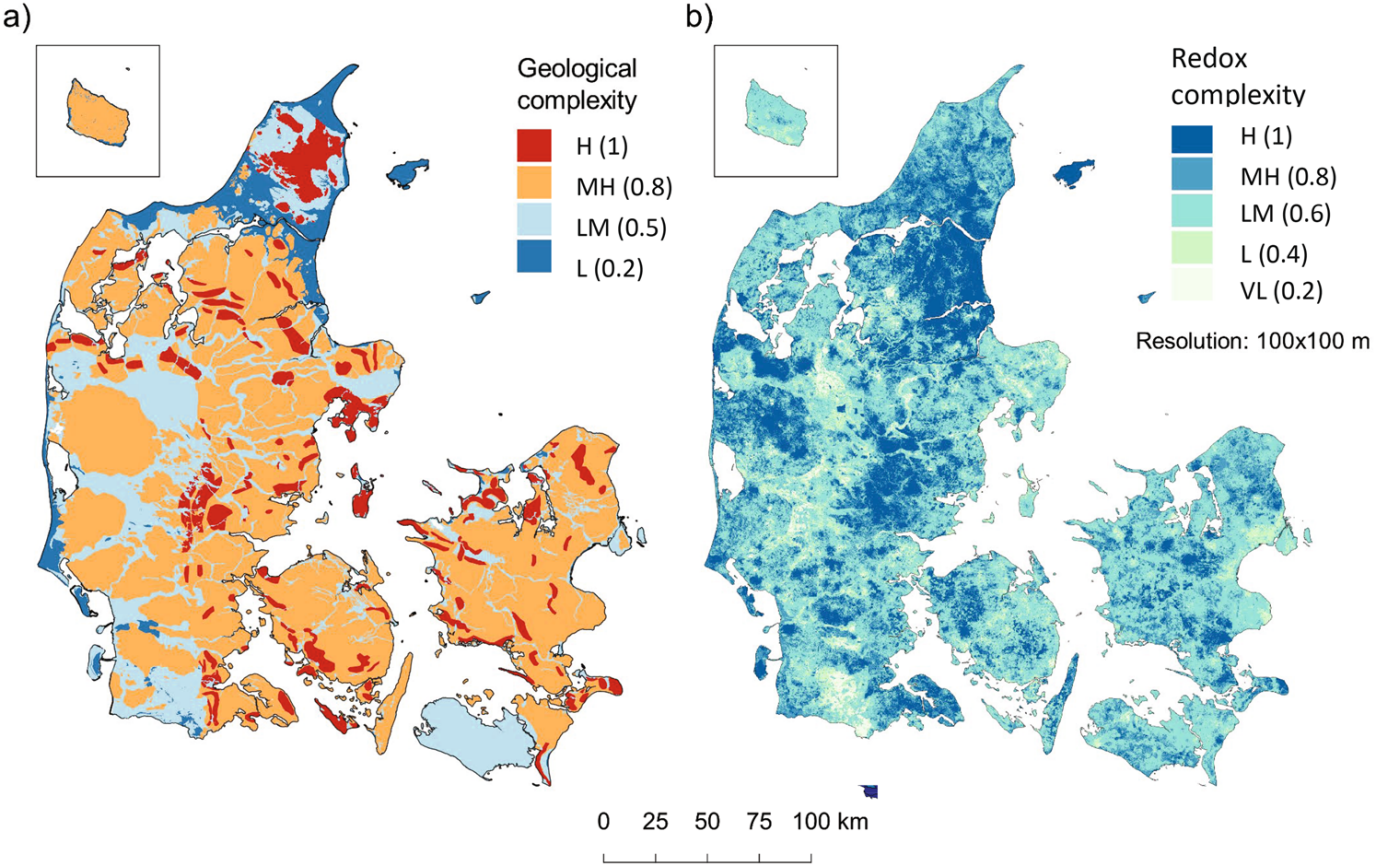
The geological and redox complexity in Denmark. Complexity is described as high (H), medium high (MH), low medium (LM), low (L) and VL (very low). Scoring of each of the maps are shown in parentheses from 0.2 (low complexity) to 1 (high complexity). Generated with QGIS v. 3.22.10 Bialowieza^66^.

A prioritisation tool for implementing the N-MAP concept has been developed for all of Denmark based on subsurface geological and redox complexity as defined earlier. The geological complexity has been assessed based on a review of geological data and interpretations of Danish landscape elements resulting in a low-resolution map for the upper 30 m (Fig. 3a)^36^. Highly geologically complex areas are seen in young glacial landscapes close to ice margins where e.g., glaciotectonic deformations are abundant. Estimation of the redox complexity has been assessed based on a comparison of the colour descriptions of the sediment and groundwater nitrate concentrations sampled from boreholes (Fig. 3b)^34^. This comparison has been carried out based on two machine learning generated national maps, i.e., depth to FRI and depth to top screen of nitrate containing boreholes. The complex redox areas are to some extent coinciding with the highly geologically complex areas and are often related to older weathered glacial deposits.

**Figure 4:**
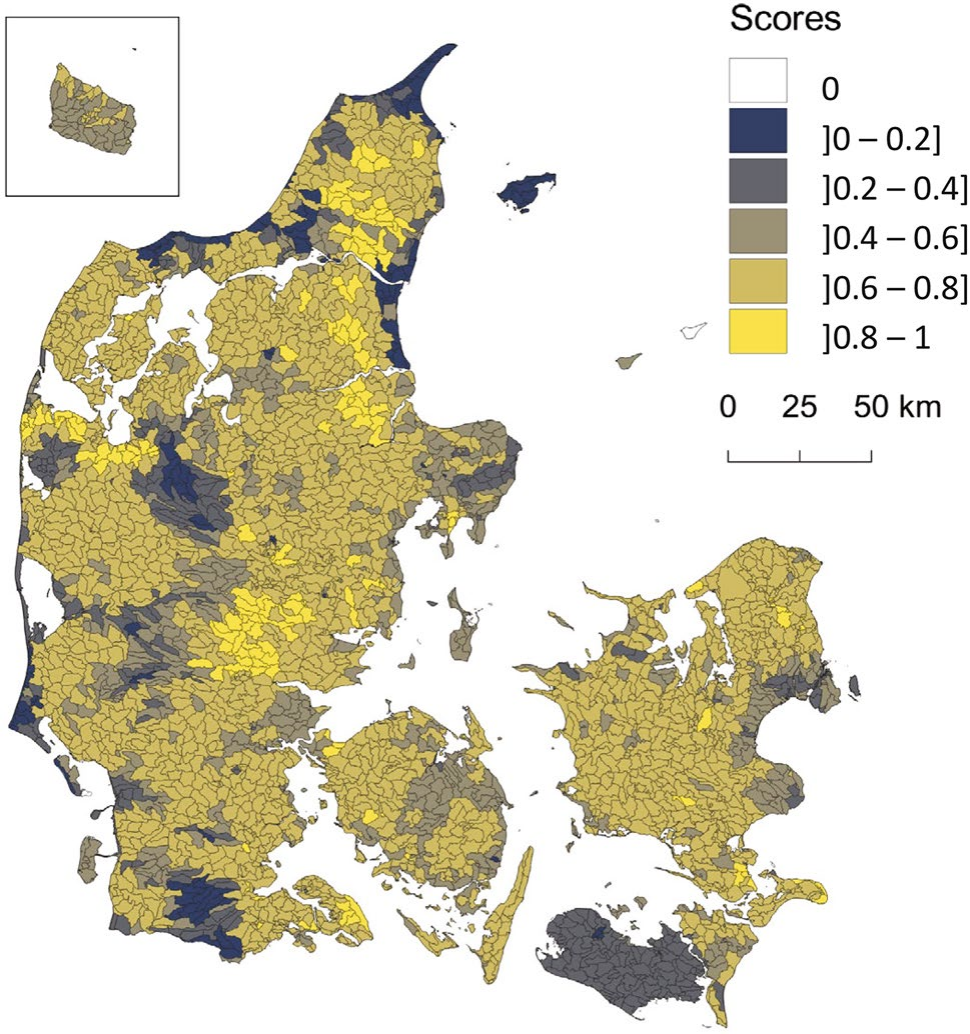
Map combining geological and subsurface redox complexity for prioritizing areas for implementing target N regulation by use of the N-MAP concept for assessment of detailed groundwater N retention in Denmark. Low priority areas have a low score, and high priority areas have a high score. Generated with QGIS v. 3.22.10 Bialowieza^66^.

The prioritisation tool has been developed at catchment scale (*c.* 15 km^2^) as a composite indicator^39^ by combining the two indicators of geological and redox complexity shown in Fig. 3. The indicator maps of geological and redox complexity are rated gradually in the range from 0 to 1 as shown in Fig. 3. In Fig. 4, the two maps in Fig. 3 have been multiplied and high-priority catchments are shown with the highest score (bright areas), and low-priority catchments with a low score (dark colors). A high geological and redox complexity (score 0.8–1) results in a score from 0.64 to 1 in the prioritisation tool shown in Fig. 4, which covers 70% of the agricultural fields in Denmark. Prioritisation solely of areas with the most complex subsurface structures (score 0.8 and 1) would lower N-MAP deployment costs considerably because these areas only cover 32% of Danish agricultural fields.

The focus in this paper is on the importance of subsurface complexity for groundwater N retention. However, further prioritisation can be performed for example by deselecting catchments where the drainage transport is a controlling factor for low N retention, where the N retention mainly takes place in the surface water system, and where there is no current N reduction demands on the coastal areas.

## Economic benefits with new targeted N regulation

The economic gain from targeted N regulation depends on many factors such as N reduction demands, the choice of mitigation measures, and the scale of regulation. The Danish Economic Council has found that targeting of agricultural N mitigation measures from national to catchment level can reduce the costs of achieving the goals of the EU's Water Framework Directive significantly^40^. Other analyses have shown that further targeting from catchment to field level could reduce costs by another 20–30%^14^.

An assessment of the potential economic gain for the farmers in the demo sites in northwestern Denmark (Fig. 2) has been performed based on interviews with these farmers where e.g., soil type, crop rotation, animal density and drainage were considered. Two different detail levels were selected: (1) the 250 × 250 m targeted level obtained by use of the N-MAP concept, and (2) catchment level (c. 3000 hectares) with one retention value for the whole catchment (baseline). The recent regulatory N reduction demands for the demo sites varies a lot from the eastern to the western part of the area^41^. The eastern part, discharging to the coastal area of Skive Fjord, has a very high N reduction demand at 722 tons N or 48% in year 2027. On the other hand, the western part only has a N reduction demand of 11 tons N or 1% in year 2027. Therefore, two different scenarios were defined for discussion with the farmers: (1) 8% N reduction, and (2) 20% N reduction to the sea, and the mitigation measure “set-aside” was chosen as an option. In scenario 1, only 5% of the area had to be set-aside using the detailed N retention maps in Fig. 2 compared to 10% if the catchment had one value for N retention for the whole catchment (baseline). In scenario 2, 15% had to be set-aside using N-MAP compared to 27% in the baseline situation. The cost reduction using the N-MAP concept was €15 per ha or €6 per kg N in scenario 1, and €31 per ha or €5 per kg N in scenario 2 (Table 2).

**Table 2:** Economic evaluation of cost reduction by targeting N regulation at two scenarios with different N reduction demands in the demo sites in northwestern Denmark (see Fig. 2).

N reduction demands to the sea	Scenarios			
	Scenario 1 (8%)		Scenario 2 (20%)	
Detail level	250 × 250 m	Catchment	250 × 250 m	Catchment
Areal needed for set-a-side	5%	10%	15%	27%
Cost reduction per ha from targeting	€15		€31	
Cost reduction per kg N from targeting	€6		€5	

## Conclusion

The conclusion is that for this study, the expected economic gains from implementing targeted N regulation using the N-MAP concept are likely to be higher than the deployment costs. However, this might not be the case in all catchments due to different complexity in the subsurface structures (Fig. 2), degree of drainage, and legislative demands for N reduction in the affected coastal area. In addition to these economic gains on a micro level, the N-MAP concept can contribute to further societal benefits on a macro level due to e.g. reduction of N pollution of groundwater and drinking water, and reduction of health-related costs^42^.

To our knowledge, the Danish N-MAP concept is the most detailed developed concept for groundwater denitrification potential assessment relying on dense data collection and complex modelling approaches. In other parts of the world index-based methods are often applied with different versions of DRASTIC for vulnerability assessment of aquifers ^43–47^(e.g.).

Prioritisation of high-potential areas on a national level is needed to cost-efficiently implement targeted N regulation at field level informed by the new knowledge available via the N-MAP concept. The N-MAP concept in combination with the presented prioritisation tool can be adjusted and implemented in other countries with a complex geology and a need for targeted N regulation of agriculture to meet the reduction goals of the aquatic environment.

## Methods

### The N-MAP concept

The technologies and modelling tasks in the N-MAP concept were described in detail by Christiansen et al. 2023^33^. Here we summarise these methods and any modifications made.

The N-MAP concept consists of four phases: (1) compiling existing data and information, (2) high resolution geophysical mapping, geochemical sampling, and geostatistical modelling, (3) root zone modelling, groundwater flow modelling and particle tracking, and finally estimation of N retention and (4) dissemination of N retention maps to stakeholders such as farmers and authorities.

#### Phase 1: Compiling existing data and information

In phase 1 existing geological, geophysical, geochemical, hydrological, and agronomical data and information are compiled. The aim is firstly to decide if it is viable to continue with the subsequent tasks. For example, if the analysis shows that the area is heavily drained with a small groundwater contribution to surface waters then the decision may be taken to not continue with the full implementation of the N-MAP concept. Secondly, the aim is to prepare and compile existing data sources for the different modelling tasks in phases 2 and 3.

#### Phase 2: High resolution geophysical mapping, geochemical sampling, and geostatistical modelling

The first task in phase 2 is the high resolution (< 10 m laterally and a few meters vertically to 70–100 m depth) geophysical mapping of the subsurface with a dense area coverage using a recently developed towed-transient electromagnetic system (tTEM)^48–50^. The tTEM data are processed^51^, and any data affected by electromagnetic interference from man-made infrastructure are removed. A quasi-3D resistivity model is constructed by inversion of the tTEM data using a 1D spatially constrained inversion (SCI) algorithm^52^ favouring sharpness (i.e. blockyness)^53^.

The second task in phase 2 is to sample and analyse water and sediments to determine the 3D redox structures and denitrification rates in the groundwater. The redox conditions are interpreted based on the groundwater concentrations of elements sensitive to redox, such as nitrate, sulphate and iron, plus sediment colours and Fe(II)/Fe(total) of formic acid sediment extracts.^35,37^ Redox conditions are divided into three zones as follows:

Oxic zone:

- Sediment colors: yellow, red, brown and combinations of these “oxic” colours
- Water chemistry: consistently high nitrate concentrations and low (non-elevated) sulphate concentrations derived from infiltration.
- Sediment chemistry: close to zero to very low Fe(II)/ Fe(total) fraction in formic acid extracts

Anoxic nitrate-reducing zone:

- Sediment colours: grey, green, olive; mixed colours related to oxic and reduced conditions
- Water chemistry: low or decreasing nitrate concentrations and often increasing sulphate concentrations with increasing depth due to pyrite oxidation
- Sediment chemistry: increasing Fe(II)/Fe(total) fraction.

Reduced zone:

- Sediment colours: grey, green, olive and combinations of these “reduced” colours
- Water chemistry: nitrate concentrations below 1 mg/l
- Sediment chemistry: Fe(II)/Fe(total) fraction close to 1

The denitrification rates in the anoxic nitrate reducing zone and the reduced zone are determined in the laboratory using the core samples collected from the study catchment by the acetylene-blockage method^54^. In oxic zones, where denitrification is assumed to be negligible, the denitrification rate is set to zero^55^.

The third task in phase 2 is the integrated 3D modelling of hydro-stratigraphy and N reduction zones^56^. Here the construction of hydro-stratigraphical structures is based on the accumulated clay thickness (ACT) method^57^ followed by a k-means cluster analysis^58^. The multiple point geostatistical simulation method of Direct Sampling (DS) is used to capture and describe relevant uncertainties across the model domain by generating many (500) equiprobable model realisations^59^. The training image used in DS is generated directly from the hydrostratigraphical units output from the clustering. The N reduction model is produced by synthesising interpretations of the redox zones and N reduction rates based on the water and sediment sample analyses, the tTEM resistivity models, and the interpretation of geological structures. Multiple realisations of the N reduction model are co-simulated with the hydro-stratigraphical units by DS using the upscaled conceptual redox-rate model as the training image^55^, which is a modification of the first version of the N-MAP concept presented in Christiansen et al. ^30^.

#### Phase 3: Root zone modelling, groundwater flow modelling and particle tracking

Phase 3 uses a script-based modelling framework. Nitrate leaching in the root zone is estimated using the empirical NLES5 model^60^, and percolation is estimated by the root zone mechanistic and deterministic model Daisy^61^. The NLES5 model is partly based on data from the Agricultural Catchment Monitoring Programme^62^, and requires input data for agricultural practices (N fertilisation, cropping system), soil data and water percolation from the root zone. The Daisy model requires daily climate data from a 10 km grid net that covers Denmark, representing the period 1991–2016. These data were obtained from the Danish Meteorological Institute.

The 3D, physically-based, fully-distributed groundwater flow model, MODFLOW6 is used to simulate groundwater flow from the water table to surface water^63^, and particle-tracking is modelled by MODPATH7^64^. The steady-state MODFLOW model was set-up for the period from 1990/91 to 2009/ 10. The model was set up on a 30 m grid and had 10 layers: 2 m thick for layers 1–5 and 10 m thick for layers 6–10. The structural input to the groundwater model is a realisation of the hydro-stratigraphical units derived from DS. Each unit is then populated with a parameter-set drawn from an estimated probability distribution for each parameter. For the particle tracking, a particle is released in each water table cell in the groundwater model carrying a nitrate concentration estimated using NLES5.

The potential denitrification in groundwater, $N_{redpot,GW} ,$ along a single particle flow path is calculated as:

1 $$N_{{redpot,GW{ }}} = \mathop \sum \limits_{i = 1}^{n} \tau_{i} \frac{dC}{{dt_{i} }}$$

where $\tau_{i}$ is the transit time through redox zone $i$ with denitrification rate $\frac{dC}{{dt_{i} }}$ cumulated over $n$ redox zones along the flow path. The transit time through a denitrification zone is a function of particle velocity and flow path through the zone. The particle velocity is controlled by hydrogeological parameters such as hydraulic conductivity and effective porosity, while the flow path is controlled by hydrogeological structures.

N retention in groundwater is calculated as:

2 $$N_{ret,GW} = \frac{{N_{redpot,GW} }}{{N_{leaching} }}$$

where $N_{leaching}$ from the root zone was estimated using NLES5.

While $N_{redpot,GW}$ is invariant over time, $N_{{leaching{ }}}$ varies temporally. However, for this study, $N_{{leaching{ }}}$ is assumed constant, and so we use the average leaching of N out of the root zone for a 20-year period for the entire catchment as input for the calculation of groundwater N retention.

#### Phase 4: Dissemination of N retention maps to stakeholders

In phase 4, the N retention maps are presented for stakeholders in a specific catchment. The groundwater N retention maps at field level as well as the total nitrogen retention are presented to farmers and advisors. The maps are also discussed with relevant authorities regarding current and future legislation as well as possible strategies for the implementation of more targeted nitrogen regulation.

### The prioritisation tool

The prioritisation tool for implementation of the N-MAP concept in Denmark consists of the two themes—a geological complexity theme and a redox complexity theme based on the ideas of Hansen et al.^35^, Sandersen^36^, and Voutchkova et al.^34^. The prioritisation tool was developed as a composite indicator formed by compiling individual indicators in a combined single index, reflecting a complex 2D system^39^. The derivation of the prioritisation map can be represented by the following equation:

3 $$Prioritization\, tool = \mathop \prod \limits_{i = 1}^{2} T_{i} = \left( {T_{1} *T_{2} } \right)$$

where *T*_*i*_ are the two rated thematic maps (geological and redox complexity) and *i* is an index for each of them. The multiplication procedure is carried out in a GIS environment (QGIS v. 3.10).

#### Theme 1: Geological complexity

**Figure 5:**
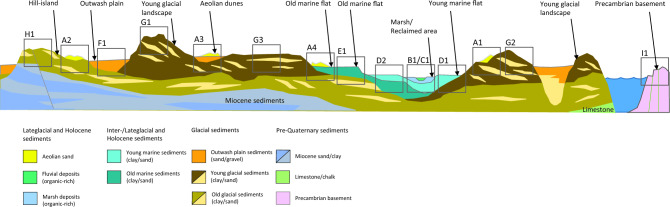
Classification of landscape types^36^.

A high geological complexity within a catchment is expected to give a high variation in the groundwater flow paths. This is anticipated to give a high variation in the N retention maps within the catchment, and thereby offering a potential for a differentiated field-scale N regulation. The GIS map of geological complexity is based on landscape types in the upper 30 m of subsurface^36^. Figure 5 shows the classification of the different landscape types, while Table 3 shows their complexity.

**Table 3:** Classification of the landscape types^37^.

Unit	Sub-unit	Landscape type	Complexity
A	1	Aeolian dune on young glacial landscape	MH
	2	Aeolian dune on hill-island	MH
	3	Aeolian dune on outwash plain	L
	4	Aeolian dune on Late- or Postglacial marine flat	L
B	1	Reclaimed area (former lake or marine fiord)	LM
C	1	Marsh on older flats (marine/non marine)	LM
D	1	Young marine flat (Littorina) on older sediments (glacial/non glacial)	LM
	2	Young marine flat (Littorina) on older marine flat (Yoldia)	L
E	1	Old marine flat (Yoldia) on older sediments	LM
F	1	Outwash plain on older sediments	LM
G	1	Young glacial landscape, ice-margins, on older landscape/sediments	H
	2	Young glacial landscape, hilly, on older landscape/sediments	MH
	3	Young glacial landscape, smooth, on older landscape/sediments	LM
H	1	Old glacial landscape (hill-island) on older landscape/sediments	MH
I	1	Precambrian basement	L

L, low complexity; LM, low to moderate complexity; MH, moderate to high complexity; H, high complexity.

All 15 landscape types were separated into individual GIS layers, which were then aggregated into four layers of variable geological complexity as follows: low (L), low to moderate (LM), moderate to high (MH), and high (H). In this way, all areas were assigned to one of the four complexity classes. Then, using the QGIS tool “Overlap analysis”, the percentage of the area in each catchment was calculated covered by each of the four complexity types (L, LM, MH, H). Each catchment was then classified in one of the four classes, based on the highest complexity class as seen in Fig. 3.a. The low geological complexity (L) was assigned a ‘very low’ score (0.2) and the low to moderate complexity (LM) a ‘medium’ score (0.5). The moderate to high (MH), and high (H) complexity groups were given ‘high’ and ‘very high’ scores of 0.8 and 1, respectively.

#### Theme 2: Redox complexity

A high redox complexity within a catchment indicates a high variation in the groundwater flow paths and in the groundwater nitrate reduction. This is anticipated to give a high variation in the N retention maps between fields within the catchment, and thereby the potential for differentiated N regulation on the fields.

The complexity of the redox structures was analysed by comparing two machine learning generated maps, i.e., the depth of the nitrate-containing groundwater and the depth of the first redox interface (FRI)^22^. The first was trained against interpreted sediment colour data, whereas the second was trained against analysed water chemistry data. The presence of nitrate-containing water below the FRI was defined as an indication of complex groundwater flow paths and complex nitrate reduction processes in underlying geological layers below the FRI. Agreement between the FRI and the depth of the nitrate-containing groundwater (< 10 m difference), indicated very low or low redox complexity and a low priority for implementation of N-MAP. On the other hand, the larger the difference between the two maps, the stronger indication of redox complexity, and therefore a higher priority for implementing N-MAP. In the highest redox complexity class, the difference exceeds 25 m (dark blue areas in Fig. 7).

**Figure 6:**
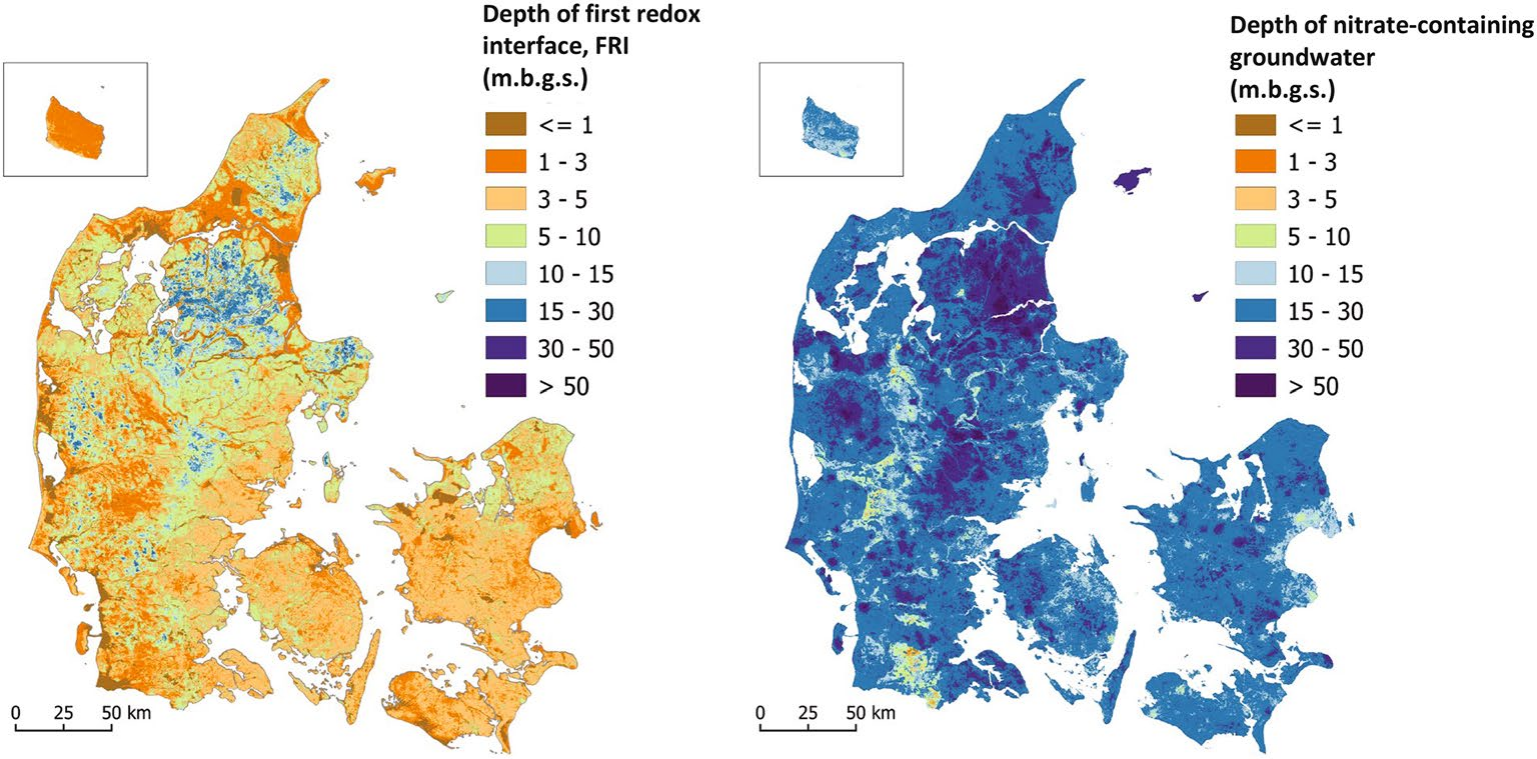
Input data for Theme 2: First redox interface, FRI^22^, and modelled depth of nitrate-containing groundwater (top intake); m.b.g.s. is meters below ground surface. Generated with QGIS v. 3.22.10 Bialowieza^66^.

Data sources for Theme 2 included (1) the national FRI map^22^ and (2) a map showing the depth to nitrate-containing groundwater (Fig. 6).

Nitrate-containing groundwater was mapped by applying the same machine learning modelling approach used for the FRI map^22^, on the depth of well-screens containing nitrate. This depth was based on the top of the deepest nitrate-containing well-screen (> 1 mg/l).

The modelling was done in two steps: Two Random Forest (RF) models were trained using well data and 18 covariates ^22^. The training data sets contained 15,601 wells for the first redox interface (FRI map) and 3,167 wells for modelling the depth to nitrate-containing groundwater based on the screen depth, and water chemistry data. The 18 covariates comprised national maps, resampled to 100 m, reflecting the geological, hydrological, and topographical variability. After training, the two models were used to predict the variables at a spatial resolution of 100 m. For the FRI map, two geostatistical models (hill-islands area and the rest of the country) of the RF residuals were built by fitting a variogram model that was subsequently applied in a Kriging interpolation. For more details we refer to Koch et al.^22^. To map the depth of nitrate-containing groundwater, a single geostatistical model covering the entirety of Denmark was built to interpolate the RF residuals. Finally, the interpolated residuals were added to the RF prediction. This method is referred to as Random Forest Regression Kriging.

**Figure 7:**
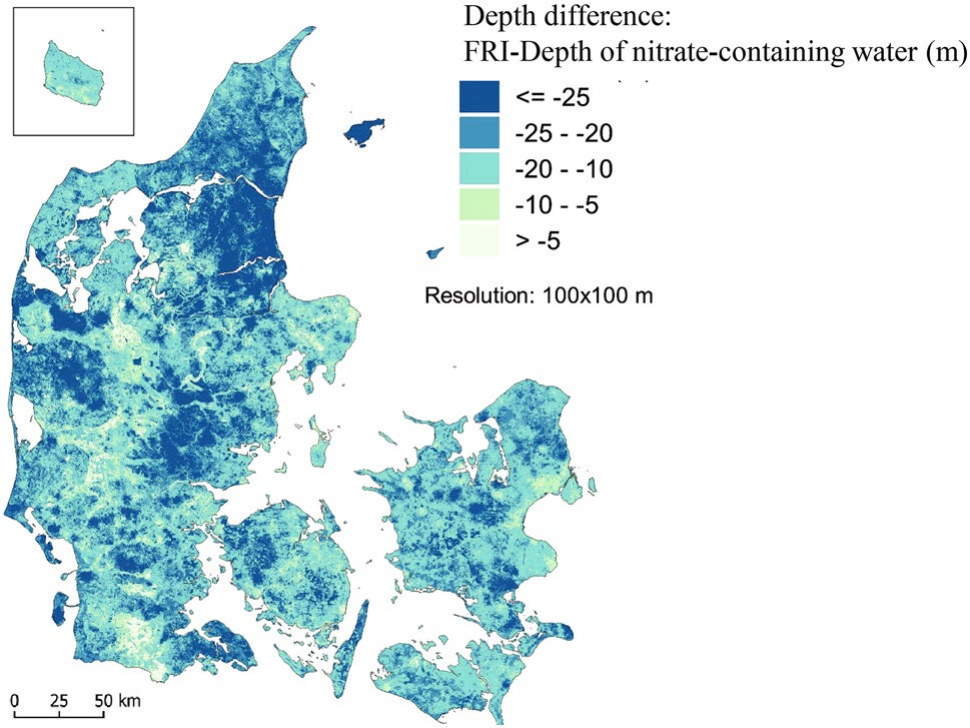
Depth difference between the first redox interface (FRI) and the depth to nitrate-containing groundwater. Generated with QGIS v. 3.22.10 Bialowieza^66^.

To derive the map for Theme 2, the two raster maps from Fig. 6 were subtracted from each other (FRI–depth of nitrate-containing water) and then the median depth difference for each catchment was calculated (Fig. 7).

The scoring of Theme 2 (redox complexity) shown in Fig. 7 was evaluated. Subsequently it was decided to use a scoring that gradually increased with increased depth-difference. The lowest score (0.2) was given to the catchments where the difference between the first redox interface and the depth to nitrate-containing groundwater was less than 5 m. The highest score (1) was given to the ID15 catchments where the difference was larger than 25 m.

In Fig. 3b the five levels of redox complexity shown in Fig. 7 was categorised in the same way as the map of geological complexity shown in Fig. 3a. The very low redox complexity (VL) was assigned a ‘very low’ score (0.2), low complexity was assigned a ‘low’ score (0.4) and the low to moderate complexity (LM) was assigned a ‘medium’ score (0.6). The moderate to high (MH), and high (H) complexity groups were given ‘high’ and ‘very high’ scores of 0.8 and 1, respectively.

## Data Availability

The datasets generated and/or analysed during the current study are not publicly available due to the research collaboration agreement between the co-authors but are available from the corresponding author on reasonable request.
